# Topology, Cross-Frequency, and Same-Frequency Band Interactions Shape the Generation of Phase-Amplitude Coupling in a Neural Mass Model of a Cortical Column

**DOI:** 10.1371/journal.pcbi.1005180

**Published:** 2016-11-01

**Authors:** Roberto C. Sotero

**Affiliations:** Hotchkiss Brain Institute, Department of Radiology, University of Calgary, Calgary, AB, Canada; Brain and Spine Institute (ICM), FRANCE

## Abstract

Phase-amplitude coupling (PAC), a type of cross-frequency coupling (CFC) where the phase of a low-frequency rhythm modulates the amplitude of a higher frequency, is becoming an important indicator of information transmission in the brain. However, the neurobiological mechanisms underlying its generation remain undetermined. A realistic, yet tractable computational model of the phenomenon is thus needed. Here we analyze a neural mass model of a cortical column, comprising fourteen neuronal populations distributed across four layers (L2/3, L4, L5 and L6). A control analysis showed that the conditional transfer entropy (cTE) measure is able to correctly estimate the flow of information between neuronal populations. Then, we computed cTE from the phases to the amplitudes of the oscillations generated in the cortical column. This approach provides information regarding directionality by distinguishing PAC from APC (amplitude-phase coupling), i.e. the information transfer from amplitudes to phases, and can be used to estimate other types of CFC such as amplitude-amplitude coupling (AAC) and phase-phase coupling (PPC). While experiments often only focus on one or two PAC combinations (e.g., theta-gamma or alpha-gamma), we found that a cortical column can simultaneously generate almost all possible PAC combinations, depending on connectivity parameters, time constants, and external inputs. PAC interactions with and without an anatomical equivalent (direct and indirect interactions, respectively) were analyzed. We found that the strength of PAC between two populations was strongly correlated with the strength of the effective connections between the populations and, on average, did not depend on whether the PAC connection was direct or indirect. When considering a cortical column circuit as a complex network, we found that neuronal populations making indirect PAC connections had, on average, higher local clustering coefficient, efficiency, and betweenness centrality than populations making direct connections and populations not involved in PAC connections. This suggests that their interactions were more effective when transmitting information. Since approximately 60% of the obtained interactions represented indirect connections, our results highlight the importance of the topology of cortical circuits for the generation of the PAC phenomenon. Finally, our results demonstrated that indirect PAC interactions can be explained by a cascade of direct CFC and same-frequency band interactions, suggesting that PAC analysis of experimental data should be accompanied by the estimation of other types of frequency interactions for an integrative understanding of the phenomenon.

## Author Summary

For many decades, the study of oscillatory brain activity focused on the separate analysis of its different frequency bands (from delta to gamma). However, neurons, and neuronal populations are nonlinear systems, and a sinusoidal input will produce new frequency components in their output. This induces cross-frequency coupling (CFC) between any two sources (e.g. neuronal populations, or brain regions) when there are bidirectional connections between them, as is often the case in the brain. Cascades of nonlinear sources can also produce CFC between sources that are not directly connected. Although several types of CFC are possible, there is an increasing interest in phase-amplitude coupling (PAC), the phenomenon where the amplitude of a high frequency oscillation (e.g. gamma) is modulated by the phase of a lower frequency (e.g. theta). PAC has been hypothesized to mediate the integration of distributed information in the brain, but the exact local and global mechanisms responsible for this processing remain unknown. Here we focus on the generation of PAC at the local scale, in the cortical column, and study how the biophysics of the neuronal populations involved, influence the generation of the phenomenon. Our results highlight the importance of the topology of the cortical column network on the generation of PAC, and show that indirect PAC connections can be predicted by a cascade of direct same-frequency coupling (SFC) and CFC connections.

## Introduction

It has been hypothesized that phase-amplitude coupling (PAC) of neurophysiological signals plays a role in the shaping of local neuronal oscillations and in the communication between cortical areas [1]. PAC occurs when the phase of a low frequency oscillation modulates the amplitude of a higher frequency oscillation. A typical example of this phenomenon was registered in the CA1 region of the hippocampus [2], where the phase of the theta band modulated the power of the gamma-band. Computational models of the theta-gamma PAC generation in the hippocampus have been proposed [3] and are based on two main types of models. The first type of models consists of a network of inhibitory neurons (I-I model) [4], whereas the second model is based on the reciprocal connections between networks of excitatory pyramidal cells and inhibitory neurons (E-I model) [3, 5]. In such models, fast excitation and delayed feedback inhibition alternate, and with appropriate strength of excitation and inhibition, oscillatory behavior occurs. When the gamma activity produced by the E-I or I-I models is periodically modulated by a theta rhythm imposed by either an external source or theta resonant cells within the network [4], a theta-gamma PAC is produced. Recently, the generation of theta-gamma PAC was studied [6] using a neural mass model (NMM) proposed by Wilson and Cowan [7]. In NMMs, spatially averaged magnitudes are assumed to characterize the collective behavior of populations of neurons of a given type instead of modeling single cells and their interactions in a realistic network [7, 8]. Specifically, the Wilson and Cowan model consists of excitatory and inhibitory neural populations which are mutually connected.

While the models mentioned above have improved our understanding of the physiological mechanisms that give rise to theta-gamma PAC, we lack modeling insights into the generation of PAC involving other frequency pairs [9]. This is critical because experimental studies have shown that the PAC phenomenon is not restricted to either the hippocampus or to theta-gamma interactions. In fact, PAC has been detected in pairs involving all possible combinations of low and high frequencies: delta-theta [10], delta-alpha [11, 12], delta-beta [11, 13], delta-gamma [13–17], theta-alpha [11], theta-beta [11, 13], theta-gamma [10, 15, 16, 18–21], alpha-beta [22], alpha-gamma [23–26], and beta-gamma [23, 27]. Although experimental studies usually focus on one or two PAC combinations, most of the combinations mentioned above can be detected in a single experiment [22]. Furthermore, PAC can represent indirect interactions, but modelling studies [6] have focused on PAC mediated by direct (anatomical) connections. If PAC is involved in the transmission of information between brain regions then we need to understand how indirect PAC connections are created.

The issues mentioned above suggest a diversity and complexity of the PAC phenomenon that has been overlooked by previous theoretical studies. Similarly, there is a need for further improvement in the mathematical methods used to detect PAC. Although a large number of methods have been proposed [28, 29], no gold standard has emerged.

In this work, we analyze a neural mass model of a cortical column that comprises 4 cortical layers and 14 neuronal populations [30, 31], and study the simultaneous generation of all PAC combinations mentioned above. To estimate PAC we use a measure of the information transfer from the phase of the low frequency rhythm to the amplitude of the higher frequency oscillation, which is known as conditional transfer entropy (cTE) [32]. This multivariate approach provides information about the directionality of the interactions, thus distinguishing PAC from the information transfer from the amplitude to the phases (i.e. amplitude-phase coupling, or APC) which has been experimentally detected [33]. This is done in contrast to previous methods which were either based on pairwise correlations between the selected phase and amplitude [28, 34], or provided directionality using pairwise approaches [33], or were multivariate but did not provide directionality [35]. By estimating cTE from phases to amplitudes, we obtain a clearer view of the mechanisms underlying the generation of PAC in the cortical column. Specifically, we focus on the link between anatomical and effective PAC structures. In the examples shown in this paper, the neuronal populations have natural frequencies in the theta, alpha and gamma bands. However, due to the effective connectivity between populations, oscillations in the delta and beta bands appear and result in PAC involving these frequencies. We focused on three PAC combinations (delta-gamma, theta-gamma, and alpha-gamma) and explored how changes in model parameters such as the strength of the connections, time constants or external inputs strengthen or weaken the PAC phenomenon. We found that approximately 60% of the obtained PAC interactions result from indirect connections and that, on average, these interactions have the same impact as direct (anatomical) connections. The cortical column circuit was analyzed as a complex network and three different local topological measures were computed: the clustering coefficient (*C*_*m*_), the efficiency (*E*_*m*_) and betweenness centrality (*B*_*m*_) which quantify how efficiently the information is transmitted within the network. According to our results, neuronal populations sending (receiving) indirect PAC connections had higher local *C*_*m*_, *E*_*m*_, and *B*_*m*_ coefficients, than populations sending (receiving) direct PAC connections and populations not involved in PAC interactions. This suggests that the topology of cortical circuits plays a central role in the generation of the PAC phenomenon.

Finally, although this paper focuses on the PAC phenomenon, our theoretical results suggest that in order to understand the generation of indirect PAC connections we may need to estimate other types of cross-frequency coupling such as APC, amplitude-amplitude coupling (AAC), and phase-phase coupling (PPC), as well as interactions within the same frequency band (or same-frequency coupling, SFC). We computed these measures in a simplified three-population model and used them as predictors of indirect PAC in a linear regression analysis. We demonstrated that indirect PAC connections can be predicted by a cascade of direct CFC and SFC interactions, suggesting that PAC analysis of experimental data should be accompanied by the estimation of other types of interactions for an integrative understanding of the phenomenon.

A list of the abbreviations used in this paper is presented in Table 1.

**Table 1:** List of abbreviations.

Abbreviation	Meaning
AAC	Amplitude-amplitude coupling
APC	Amplitude-phase coupling
CFC	Cross-frequency coupling
cMI	Conditional mutual information
cTE	Conditional transfer entropy
ECoG	Electrocorticography
EEG	Electroencephalography
ESC	Envelope-to-signal correlation
FS	Fast-spiking
IB	Intrinsically bursting
LFP	Local field potential
LTS	Low-threshold
Midx	Modulation index
NMM	Neural mass model
PAC	Phase-amplitude coupling
PFC	Phase-frequency coupling
PPC	Phase-phase coupling
PSP	Postsynaptic potential
RS	Regular spiking
SFC	Same-frequency coupling

## Methods

### A neural mass model of a cortical column

**Fig 1:**
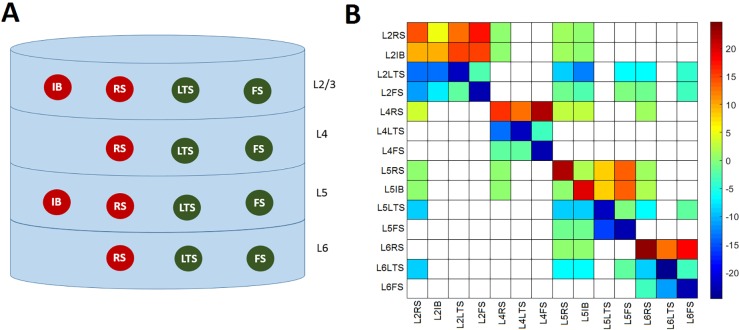
Proposed neural mass model of the cortical column. A) Layer distribution of the four neuronal types. The excitatory populations are the intrinsically bursting (IB), and the regulatory spiking (RS). The inhibitory populations are low-threshold spiking (LTS) and fast spiking (FS). B) Connectivity matrix values used for coupling the 14 populations modeled. Negative values correspond to inhibitory connections.

Fig 1 shows the proposed model obtained by distributing four cell classes in four cortical layers (L2/3, L4, L5, and L6). This produced 14 different neuronal populations, since not all cell types are present in each layer [31]. Excitatory neurons were either regular spiking (RS) or intrinsically bursting (IB), and inhibitory neurons were either fast-spiking (FS), or low-threshold spiking (LTS) neurons. We omitted layer 1, because it does not include somas [36]. Based on experimental reports on the strength of the inputs to each layer [36, 37], we only consider external inputs to the RS and FS populations in layer 4, thus neglecting possible external inputs to other layers.

The evolution of each population dynamics rests on two mathematical operations. Post-synaptic potentials (PSP) at the axonal hillock were converted into an average firing rate using the sigmoid function [8]:

$$S\left(x\right)=\frac{e_{0}}{1+e^{r\left(v_{0}-x\right)}}$$ (1)

where the variable *x* represents the PSP and parameters *e*_*0*_, *v*_0_ and *r* represent the maximal firing rate, the PSP corresponding to the maximal firing rate e_0_, and the steepness of the sigmoid function, respectively. For a more realistic model of the potential to rate transformation see [38]. The second operation was the conversion of firing rate at the soma and dendrites into PSP, which was done by means of a linear convolution with an impulse response *h*(*t*) given by:

$$h\left(t\right)=Ggte^{-gt}$$ (2)

where *G* controls the maximum amplitude of PSP and *g* is the sum of the reciprocal of the average time constant [8]. The convolution model with impulse response (2) can be transformed into a second order differential equation [8, 39]. The temporal dynamics of the average PSP in each neuronal population *x*_*m*_ is described by a system of 14 second order differential equations:

$$\frac{d^{2}x_{m}\left(t\right)}{dt^{2}}=-2g_{m}\frac{dx_{m}\left(t\right)}{dt}-g_{m}^{2}x_{m}\left(t\right)+G_{m}g_{m}\left(p_{m}+\sum_{n=1}^{14}\Gamma_{nm}S\left(x_{n}\left(t\right)\right)\right)$$ (3)

where *n* = 1,…,14 and *m* = 1,…,14. The populations are numbered from 1 to 14 following the order: [L2RS, L2IB, L2LTS, L2FS, L4RS, L4LTS, L4FS, L5RS, L5IB, L5LTS, L5FS, L6RS, L6LTS, L6FS]. Note that layer 2/3 was simply labelled as L2. As can be seen in (3), neuronal populations interact via the connectivity matrix Γ_*nm*_. This is an ‘anatomically constrained’ effective connectivity matrix [30] in the sense that its elements represent anatomical (i.e., direct) connections, but their strength (except the ones set to zero) can vary with a condition or task. External inputs to the cortical column are accounted for via *p*_*m*_, which can be any arbitrary function, including white noise [8]. Thus, Eq (3) represents a system of 14 random differential equations [40, 41]. Eq (3) is the equation of a damped oscillator with a damping parameter set to 1. In this work we generalize Eq (3) by introducing the damping parameter *b*_*m*_:

$$\frac{d^{2}x_{m}\left(t\right)}{dt^{2}}=-2g_{m}b_{m}\frac{dx_{m}\left(t\right)}{dt}-g_{m}^{2}x_{m}\left(t\right)+G_{m}g_{m}\left(p_{m}+\sum_{n=1}^{14}\Gamma_{nm}S\left(x_{n}\left(t\right)\right)\right)$$ (4)

Parameter *b*_*m*_ critically determines the behavior of the system. If the connections between the populations are set to zero (Γ_*nm*_ = 0, *n* ≠ *m*), then for *b*_*m*_ > 1 (overdamped oscillator) and *b*_*m*_ = 1 (critically damped oscillator), each neuronal population will evolve to a fixed point $\left(\frac{dx_{m}\left(t\right)}{dt}=0\right)$ without oscillating. If *b*_*m*_ < 1 (underdamped oscillator), each population is capable of producing oscillations even if the inter-population coupling is set to zero. As mentioned previously, the case *b*_*m*_ = 1 corresponds to the Jansen and Rit model [8], which has been extensively used in the literature [39, 42–48]. Thus, in [8] and the models based on it, an individual population is not capable of oscillating, and the balance between excitation and inhibition is what produces oscillatory behavior that mimics observed Electroencephalography (EEG) signals. It should be noted that realistic models of a single inhibitory neural population are able to produce oscillations [49], but that excitatory populations were believed to only produce unstructured population bursts [50]. This view has been challenged recently by both experimental and computational studies [51, 52]. To account for the possibility of oscillatory activity in single populations, we use the parameter *b*_*m*_ with values *b*_*m*_ < 1.

To numerically solve our model, we split system (4) into a system of 28 first order differential equations:

$$\begin{array}{c} \frac{dx_{m}\left(t\right)}{dt}=y_{m}\left(t\right)\\ \frac{dy_{m}\left(t\right)}{dt}=-2g_{m}b_{m}y_{m}\left(t\right)-g_{m}^{2}x_{m}\left(t\right)+G_{m}g_{m}\left(p_{m}+\sum_{n=1}^{14}\Gamma_{nm}S\left(x_{n}\left(t\right)\right)\right) \end{array}$$ (5)

While there are many different methods for solving system (5) we selected a local linearization scheme that is known to improve the order of convergence and stability properties of conventional numerical integrators for random differential equations [39].

S1 Table presents the parameters of the model and their interpretation. As shown in the table FS populations have the fastest time constants, followed by IB, RS, and LTS, in that order. S2 Table shows the standard values of the anatomically constrained effective connectivity matrix Γ_*nm*_.

### Estimation of phase-amplitude coupling

Several mathematical methods for detecting PAC have been proposed [1, 28, 29, 33, 35], but no gold standard has emerged. Although diverse, the basis for these methods is to test the correlation between the instantaneous phase of a lower frequency rhythm and the instantaneous amplitude of the higher frequency rhythm. To compute any one of these measures, signals generated with model (5) need to be band-pass filtered into different frequency bands. In this paper we use the following bands: delta (0.1–4 Hz), theta (4–8 Hz), alpha (8–12 Hz), beta (12–30 Hz), and gamma (30–120 Hz). To this end, we designed FIR filters using MATLAB’s signal processing toolbox function *firls*.*m*. To remove any phase distortion, the filters were applied to the original time series in the forward and then the reverse direction using MATLAB’s function *filtfilt*.*m* [28]. The analytic representation *y*_*mk*_(*t*) of each filtered signal *x*_*mk*_ (where *m* = 1,..,5 stands for the index of the frequency band, and *k* = 1,..,14, indexes the neuronal populations) was obtained using the Hilbert transform Hilbert(*x*_*mk*_(*t*)):

$$y_{mk}\left(t\right)=x_{mk}\left(t\right)+i\text{Hilbert}\left(x_{mk}\left(t\right)\right)=a_{mk}\left(t\right)e^{i\;\varphi_{mk}\left(t\right)}$$ (6)

where *a*_*mk*_(*t*) and *φ*_*mk*_(*t*) are the instantaneous amplitudes and phases, and *i* is the imaginary number. Amplitudes were normalized by subtracting the temporal mean and dividing the result by the temporal standard deviation to create the set of normalized band-passed signals. Normalization was done to facilitate comparison between different frequency bands.

Two examples of PAC measures frequently used in the literature are the modulation index (Midx) [34] and the envelope-to-signal correlation (ESC) [28]:

$$Midx=\left|\sum_{t}a_{nl}\left(t\right)e^{i\;\varphi_{mk}\left(t\right)}\right|$$ (7)

$$ESC=corr\left(\cos\left(\varphi_{mk}\left(t\right)\right),a_{nl}\left(t\right)\right)$$ (8)

where subindexes *m* and *n* correspond to different frequency bands and subindexes *k* and *l* correspond to different neuronal populations. However, ESC and Midx are pairwise measures of the correlation between phases and amplitudes and thus cannot detect directionality in the interaction. Measures such as cTE [32] which are based on the information transmitted between signals should provide a clearer picture of the mechanisms generating PAC than correlation-based measures.

cTE can be computed using the conditional mutual information (cMI) measure [53]. First, we define the cMI between the phase *φ*_*mk*_ and the amplitude *a*_*nl*_, given all the other phases (Φ) and amplitudes (A), *I*(*φ*_*mk*_;*a*_*nl*_|*M*), using the mutual information chain rule [53]:

$$I\left(\varphi_{mk};a_{nl}|M\right)=I\left(\varphi_{mk};a_{nl},M\right)-I\left(\varphi_{mk};M\right)$$ (9)

where *M* = [Φ, A] is a matrix comprising all phases and amplitudes in all populations, except *φ*_*mk*_ and *a*_*nl*_, *I*(*φ*_*mk*_;*M*) is the mutual information between *φ*_*mk*_ and *M*, and *I*(*φ*_*mk*_;*a*_*nl*_,*M*) is the mutual information between *φ*_*mk*_, *a*_*nl*_, and M [53]. To compute cMI we use a toolbox (http://www.cs.man.ac.uk/~pococka4/MIToolbox.html) which computes several information measures using the conditional likelihood maximization algorithm [54]. cMI does not provide information about the directionality of the coupling between phases and amplitudes, which is a problem because both theoretical [55] and experimental [33] studies indicate the possibility of an information flow from amplitudes to phases. On the other hand, cTE provides directionality by estimating the cMI between one signal (the phase in our case) and the other signal (the amplitude) shifted *δ* steps into the future. In this paper, to estimate cTE from the phase to the amplitude (denoted as ${cTE}_{\varphi_{mk}↪a_{nl}}$), we compute cMI for *N* different *δ*s and average the results [32, 56, 57]:

$$cTE_{\;\varphi_{mk}↪a_{nl}}=\frac{1}{N}\sum_{\delta =1}^{N}I\left(\varphi_{mk};a_{nl}^{\delta }|\tilde{M}\right)$$ (10)

where $a_{nl}^{\delta }$ is derived from the amplitude time series *a*_*nl*_ at *δ* steps into the future, i.e. $a_{nl}^{\delta }=a_{nl}(t+\delta )$, and $\tilde{M}$ is a matrix comprising all phases and amplitudes in all populations, except *φ*_*mk*_. In this paper we use *N* = 100. Since we use a time step of 10^−4^ s in all simulations, we are averaging the cMI up to a period of 10 ms into the future.

A significance value can be attached to any of the above measures by means of a surrogate data approach [28, 34], where we offset *φ*_*mk*_ and *a*_*nl*_ by a random time lag. We can thus compute 1000 surrogate Midx, ESC, cMI and cTE values. From the surrogate dataset we first compute the mean *μ* and standard deviation *σ*, and then compute a z-score as:

$$Z_{1}=\frac{cMI-{µ}_{1}}{\sigma_{1}}\;,\;Z_{2}=\frac{cMI-{µ}_{2}}{\sigma_{2}}\;,\;Z_{3}=\frac{cMI-{µ}_{3}}{\sigma_{3}},\;Z_{4}=\frac{cTE-{µ}_{4}}{\sigma_{4}}$$ (11)

The *p*-value that corresponds to the standard Gaussian variate is also computed. Z values satisfying |*Z*| > 1.96 are significant with *α* = 0.05. Masks of zeros (for non-significant Z values) and ones (for significant Z-values) are created and multiplied to Midx, ESC, cMI, and cTE. Finally, a multiple comparison analysis based on the False Discovery Rate [58] is performed using the computed p-values.

A problem common to all methods used for estimating PAC from real data is the lack of a universal minimal interval length that guarantee an unbiased detection of PAC in all cases. However, for simulated signals without noise, or with low levels of noise, such as the ones used here, PAC can be estimated using very short segments of data, provided the phase and amplitude time series are longer than a full cycle of the slowest frequency of interest [29]. For instance, in simulations were the slowest frequency of interest corresponds to 0.1 Hz (delta oscillation), the minimum length of the time series should be ten seconds. Additionally, we select a small step size (10^−4^ s) to have enough data points to ensure a proper estimation of cMI [59].

### Modeling indirect PAC connections

Neuronal populations can interact through direct (anatomical) connections or indirectly via paths composed of consecutive direct connections. In this section, for the sake of simplicity we will focus on three interconnected populations *y*_1_ → *y*_2_ → *y*_3_. Our goal is to analyze how the indirect connection from population 1 to population 3 $\left(cTE_{y_{1}⇝y_{3}}\right)$ is related to the direct connection from population 1 to population 2 $\left(cTE_{y_{1}\to y_{2}}\right)$, and from population 2 to population 3 $\left(cTE_{y_{2}\to y_{3}}\right)$. Note that direct connections are represented by a straight arrow (→), indirect connections by a squiggle arrow (⇝), and connections not labeled as direct or indirect (see Eq 10) by an arrow with hook (↪).

Using the mutual information chain rule (9) we write the cMI corresponding to the three connections, $I\left(y_{1};y_{3}^{\delta }|\;y_{2},y_{3}\right)$, $I\left(y_{1};y_{2}^{\delta }|\;y_{2},y_{3}\right)$, $I\left(y_{2};y_{3}^{\delta }|\;y_{1},y_{3}\right)$, as:

$$I\left(y_{1},y_{3}^{\delta }|\;y_{2},y_{3}\right)=I\left(y_{1};\;y_{2},\;y_{3},y_{3}^{\delta }\right)-I\left(y_{1};\;y_{2},\;y_{3}\right)$$ (12)

$$I\left(y_{1},y_{2}^{\delta }|\;y_{2},y_{3}\right)=I\left(y_{1};\;y_{2},y_{2}^{\delta },y_{3}\right)-I\left(y_{1};\;y_{2},\;y_{3}\right)$$ (13)

$$I\left(y_{2},y_{3}^{\delta }|\;y_{1},y_{3}\right)=I\left(y_{2};\;y_{1},\;y_{3},y_{3}^{\delta }\right)-I\left(y_{2};\;y_{1},\;y_{3}\right)$$ (14)

Substituting (13) and (14) in (12) we obtain:

$$I\left(y_{1},y_{3}^{\delta }|\;y_{2},y_{3}\right)=I\left(y_{1},y_{2}^{\delta }|\;y_{2},y_{3}\right)+I\left(y_{2},y_{3}^{\delta }|\;y_{1},y_{3}\right)+I\left(y_{1};\;y_{2},\;y_{3},y_{3}^{\delta }\right)+I\left(y_{2};\;y_{1},\;y_{3}\right)-I\left(y_{1};\;y_{2},y_{2}^{\delta },y_{3}\right)-I\left(y_{2};\;y_{1},\;y_{3},y_{3}^{\delta }\right)$$ (15)

If we average (15) over *N* different lags we obtain:

$$cTE_{y_{1}⇝y_{3}}=cTE_{y_{1}\to y_{2}}+cTE_{y_{2}\to y_{3}}+\tilde{I}$$ (16)

$$\tilde{I}=I\left(y_{2};\;y_{1},\;y_{3}\right)+\frac{1}{N}\sum_{\delta =1}^{N}(I\left(y_{1};\;y_{2},\;y_{3},y_{3}^{\delta }\right)-I\left(y_{1};\;y_{2},y_{2}^{\delta },y_{3}\right)-I\left(y_{2};\;y_{1},\;y_{3},y_{3}^{\delta }\right))$$ (17)

According to (16), the indirect connection from population 1 to population 3 (*y*_1_ ⇝ *y*_3_) can be computed as the sum of the direct connections *y*_1_ → *y*_2_ and *y*_2_ → *y*_3_ plus a term $\tilde{I}$ comprising a sum of mutual information terms. We now give *y*_1_ the interpretation of the instantaneous phase in population 1, and *y*_3_ the interpretation of instantaneous amplitude in population 3:

$$cTE_{\varphi_{1}⇝a_{3}}=cTE_{\varphi_{1}\to y_{2}}+cTE_{y_{2}\to a_{3}}+\tilde{I}$$ (18)

$$\tilde{I}=I\left(y_{2};\varphi_{1},a_{3}\right)+\frac{1}{N}\sum_{\delta =1}^{N}(I\left(\varphi_{1};\;y_{2},a_{3},a_{3}^{\delta }\right)-I\left(\varphi_{1};\;y_{2},y_{2}^{\delta },a_{3}\right)-I\left(y_{2};\;\varphi_{1},\;a_{3},a_{3}^{\delta }\right))$$ (19)

The variable *y*_2_ can have the interpretation of phase, amplitude, or even instantaneous frequency [60]. Thus, Eqs (18) and (19) generalize the idea of a cascade of PAC [10], and shows that indirect PAC can be mediated by other types of CFC. Furthermore, since there is no frequency constraint for *y*_2_, $cTE_{\varphi_{1}\to y_{2}}$ or $cTE_{y_{2}\to a_{3}}$, may represent interactions within the same frequency band (i.e, SFC). Thus, we conclude that in the cascade *y*_1_ → *y*_2_ → *y*_3_, $cTE_{y_{1}⇝y_{3}}$ can be mediated by both CFC and SFC.

### Topological properties of the cortical column network

Complex network analysis have proven useful for studying the relationship between structure and function in brain networks [61]. In this paper we are interested in studying how the topology of the connectivity matrix Γ_*nm*_ influences the PAC phenomenon. Specifically, we want to answer the question of whether the populations involved in direct and indirect PAC interactions present the same topological properties. This means we need to focus on local properties of the network instead of global ones. In this paper we are going to compute three such properties: the local clustering coefficient, the local efficiency, and the local betweenness centrality, for the sending and receiving populations involved in each direct or indirect PAC interaction.

In this section we are not going to distinguish between inhibitory and excitatory connections, and the analysis will be done to the absolute value of the connectivity matrix: *W* = |Γ_*nm*_|.

Nodes (populations) of a network can be characterized by the structure of their local neighborhood. The concept of clustering of a network refers to the tendency to form cliques in the neighborhood of any given node [62]. This means that if node *m* is connected to node *n*, while at the same time node *n* is connected to node *s*, there is a high probability that node *m* is also connected to node *s*. Let *A* = {*a*_*mn*_} be the directed adjacency matrix [63] of the network (*a*_*mn*_ = 1 when there is a connection from *m* to *n*, *a*_*mn*_ = 0 otherwise). Let also $d_{m}^{tot}$ be the total degree of node *m*, and $d_{m}^{↔}=\sum_{m\neq n}a_{mn}a_{nm}$. The local clustering coefficient of node *m* for weighted networks is [64]:

$$C_{m}=\frac{{\left(\hat{W}+{\hat{W}}^{T}\right)}_{mm}^{3}}{2\left[d_{m}^{tot}\left(d_{m}^{tot}-1\right)-2d_{m}^{↔}\right]}$$ (20)

where $\hat{W}=W^{1/3}$, and ${\left(\hat{W}+{\hat{W}}^{T}\right)}_{mm}^{3}$ is the *m*th element of the main diagonal of ${\left(\hat{W}+{\hat{W}}^{T}\right)}^{3}$.

The second measure we are going to compute is the local efficiency, calculated as [65, 66]:

$$E_{m}=\frac{1}{N-1}\sum_{j,j\neq m}{\left({\vec{l}}_{mj}\right)}^{-1}$$ (21)

where ${\vec{l}}_{mj}$ is the shortest weighted path length from *m* to *j*. Thus, *E*_*m*_ is inversely related to the path length, and measures how efficiently the network exchanges information on a local scale.

To account quantitatively for the role of nodes that can be crucial for connecting different regions of the network by acting as bridges, the concept of betweenness centrality was introduced [67]. The local weighted betweenness centrality of node *m* is computed as [66]:

$$B_{m}=\frac{1}{\left(N-1\right)\left(N-2\right)}\sum_{\begin{array}{c} h,j\\ j\neq m,h\neq m,j\neq h \end{array}}\frac{\rho_{hj}\left(m\right)}{\rho_{hj}}$$ (22)

where *ρ*_*hj*_ is the number of shortest paths between *h* and *j*, and *ρ*_*hj*_(*m*) is the number of shortest paths between *h* and *j* that pass through *m*. A node with high centrality is thus crucial to efficient communication.

To compute *C*_*m*_, *E*_*m*_, and *B*_*m*_, we use Matlab functions provided in the brain connectivity toolbox (https://sites.google.com/site/bctnet/).

### Nonlinear correlation coefficient

Given the nonlinear nature of the PAC phenomenon, studying the link between the parameters of the model and the strength of PAC cannot be done only with the Pearson correlation coefficient, which measures the linear correlation between two variables. Nonlinear measures are also required. The underlying idea is that if the value of the variable *Y* is considered as a nonlinear function of the variable *X*, the value of *Y* given *X* can be predicted according to a nonlinear regression [68]. In this paper, we computed the nonlinear regression by fitting the vector *Y* of PAC values with a Fourier series:

$$\hat{Y}\left(X\right)=a_{0}+\sum_{k=1}^{K}a_{k}sin\left(b_{k}X+c_{k}\right)$$ (23)

where *K* = 10 and X is the vector of parameters. The nonlinear correlation coefficient *r*_*nl*_ is then the value of the linear correlation between *Y* and the predicted signal $\hat{Y}$.

## Results

### Detecting PAC: control analysis

**Fig 2:**
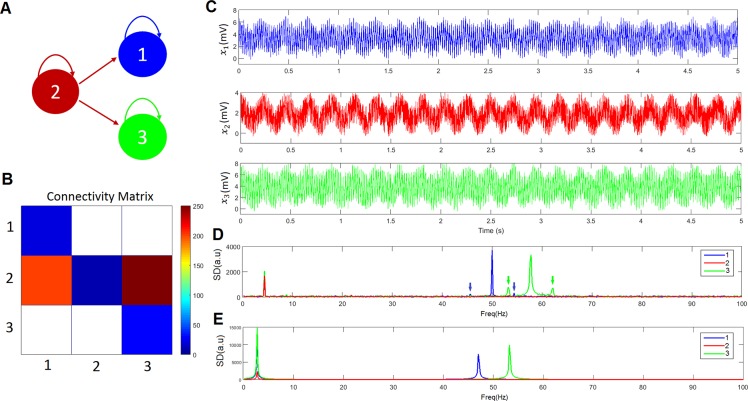
Three population toy model. A) The model comprises three neuronal populations labelled as ‘1’, ‘2’, and ‘3’, coloured in blue, red and green, respectively. This color legend is used across all panels in the figure. B) Connectivity matrix. C) Temporal dynamics of the three neuronal populations. D) Spectral density. The low frequency (4.40 Hz) is modulating the higher frequencies (50 and 57.8 HZ) which is demonstrated by the appearance of secondary peaks at frequencies 50*Hz* ± 4.40 *Hz* and 57.8*Hz* ± 4.40 *Hz* on both sides of the main peaks. The secondary peaks are indicated with arrows. E) Spectral density when substituting the sigmoid function with the linear function *S*(*x*) = *x*.

We connected three excitatory neuronal populations, labeled 1, 2 and 3 (Fig 2A and 2B). The temporal dynamics of the three populations are described by a system of random differential equations identical to (5), but with *n* = 1:3 and *m* = 1:3. As shown in Fig 2A, there is no connection between populations 1 and 3 and both are driven by population 2. The parameters used in this simulation can be found in S3 Table. Simulated data were generated by numerically integrating system (5) using the local linearization method for random systems [69] with an integration step of 10^−4^ s.

Fig 2C shows the temporal dynamics of the three populations and Fig 2D displays the corresponding spectral density. Population 2 oscillates at 4.40 Hz (theta band), whereas populations 1 and 3 have peaks at 50 and 57.8 Hz, respectively (gamma band). Because of the connections 2→1 and 2→3, there are peaks at 4.40 Hz in populations 1 and 3, and more importantly, there are secondary peaks at frequencies 50*Hz* ± 4.40 *Hz* and 57.8*Hz* ± 4.40 *Hz* on both sides of these main peaks. This shows that the low frequency (4.40 Hz) is modulating the higher frequencies (50 and 57.8 Hz) and that there is theta-gamma PAC. According to the connections shown in Fig 2A, phases in populations 1 and 3 cannot modulate the amplitudes in populations 3 and 1, respectively. Thus, an appropriate method to study the generation of PAC should not detect any modulation between populations 1 and 3. We found that when the sigmoid function is replaced by the linear function *S*(*x*) = *x*, no modulation is obtained (Fig 2E) which is consistent with the known fact the cross-frequency coupling can only be the result of nonlinear interactions. Note that in our model the only source of nonlinearities is the sigmoid function.

**Fig 3:**
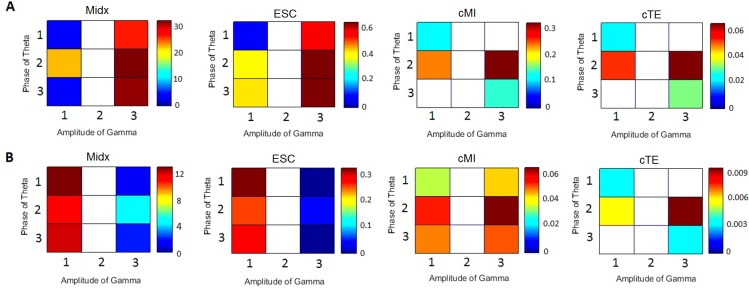
Different measures of PAC. A) Midx, ESC, cMI and TE. B) Midx, ESC, cMI and cTE when the noise is increased (*σ*_1_ = *σ*_2_ = *σ*_3_ = 10).

Fig 3A shows the PAC computed using the four measures presented in the Methods section (Midx, ESC, cMI, cTE). Non-significant values are plotted in white. The four methods correctly detect that there is no PAC involving amplitudes in the gamma band in population 2 (there is no significant spectral peak at the gamma band, only noise). However, according to ESC and Midx, there is significant PAC between the phases of the theta band in neuronal population 1 and the amplitudes of the gamma band in neuronal population 3, as well as PAC between the phases of the theta band in neuronal population 3 and the amplitudes of the gamma band in neuronal population 1. These results are expected because the signals in populations 1 and 3 are correlated, despite the fact that there is no connection between these populations. Regardless, cMI and cTE distinguished the correct effective interactions between the three populations.

There are cases where cMI fails to estimate the correct connections. For instance, Fig 3B shows the results of increasing the noise (*σ*_1_ = *σ*_2_ = *σ*_3_ = 10 *s*^−1^), which caused ESC, Mid*x* and cMI to yield similar results and estimate a significant effective connection between populations 1 and 3 that did not exist. Regardless, cTE was still able to distinguish the correct pattern of connections despite the increase in the noise level. When we further increased the noise (*σ*_1_ = *σ*_2_ = *σ*_3_ = 30 *s*^−1^), no significant results were obtained for any of the four measures (not shown in the figure).

### Generation of multiple PAC combinations

In this section, we study the generation of PAC in the cortical column circuit depicted in Fig 1. Since we are interested in the interaction between the rhythms produced by the nonlinear dynamics of the neuronal populations (not their correlation) and in the directionality of that interaction (from phases to amplitudes), we only compute cTE. The values of the parameters used are shown in S1 Table and S2 Table. Twelve seconds of data were simulated and the first two seconds were discarded to avoid transient behavior. Thus, subsequent steps were carried out with the remaining ten seconds.

**Fig 4:**
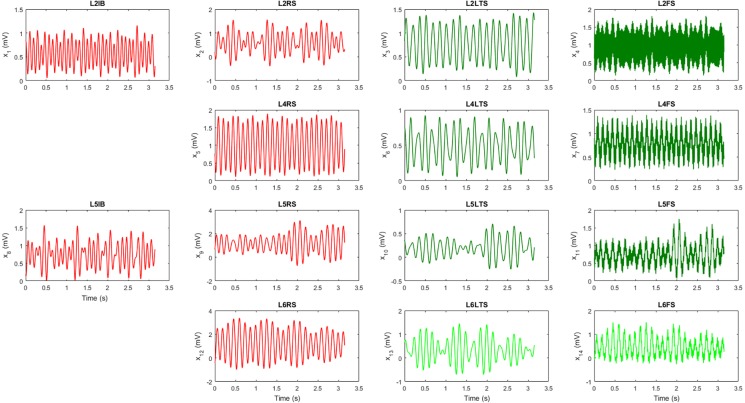
Simulated temporal evolution of the postsynaptic potentials for all populations for one realization of the model. Excitatory populations are depicted in red and inhibitory ones in green.

Fig 4 presents the temporal evolution of the average PSP in each neuronal population. For visualization purposes we show only three seconds of data. Time series coloured in red correspond to excitatory populations (L2RS, L2IB, L4RS, L5RS, L5IB, L6RS), whereas inhibitory populations (L2LTS, L4LTS, L5LTS, L6LTS) are represented in green. As seen in the figure, the generated signals show the characteristic ‘waxing and waning’ (i.e, amplitude modulation) observed in real EEG signals.

**Fig 5:**
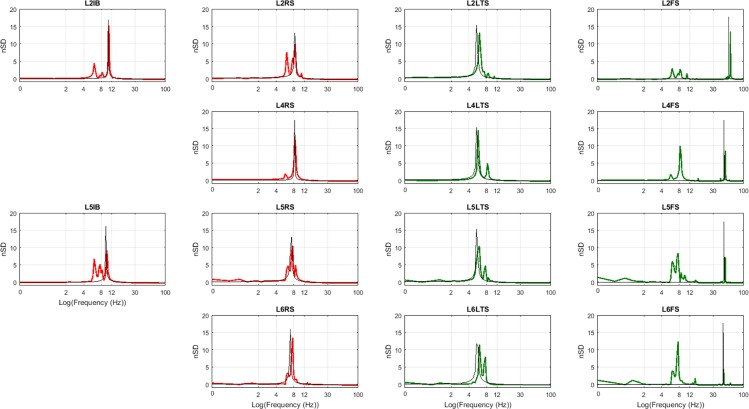
Normalized spectral density (nSD) of the postsynaptic potentials shown in Fig 3 obtained by subtracting the mean of the spectral density vector and dividing by the standard deviation. Excitatory populations are depicted in red and inhibitory ones in green. nSD coloured in black show the results when the connections between populations are set to zero.

Fig 5 presents the normalized spectrum of the average PSP in each neuronal population. Excitatory populations are depicted in red and inhibitory populations are depicted in green. The six excitatory populations have their main spectrum peak in the alpha band, but they also present energy in the delta and theta bands. Slow inhibitory populations have the highest peak in the theta band, but also have energy in the delta and alpha bands. Fast inhibitory populations were set to yield a peak in the gamma band, but due to the interaction with other populations they yield significant peaks in other frequencies as well, especially in the theta and alpha bands. This is evident when compared to the spectrum (in black) of the population when interactions between different populations are set to zero (Γ_*nm*_ = 0,*n* ≠ *m*). Peaks in black correspond to the natural frequency of oscillation for the populations: L2IB (10.48 Hz), L2RS (8.25 Hz), L2LTS (5.40 Hz), L2FS (55.87 Hz), L4RS (8.25 Hz), L4LTS (5.40 Hz), L4FS (46.35 Hz), L5IB (9.52 Hz), L5RS (7.30 Hz), L5LTS (5.40 Hz), L5FS (46.35 Hz), L6RS (6.98 Hz), L6LTS (5.40 Hz), and L6FS (44.76 Hz). Note that some peaks in the beta band are harmonics of theta and alpha oscillations, such as the beta peak at 16.83 Hz in the spectrum of L4FS in Fig 5.

**Fig 6:**
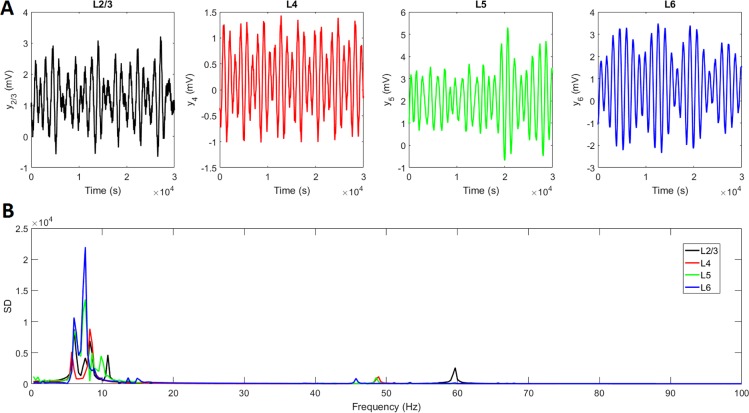
Laminar distribution of average LFP. A) Temporal dynamics in layers 2/3 (L2/3), 4(L4), 5(L5) and 6(L6). B) Spectral density (SD).

To check whether our simulations give biologically plausible results, we computed the average local field potential (LFP) in each layer as the sum of the excitatory activities in the layer [31]. Fig 6A displays the temporal dynamics of the LFP in each cortical layer and Fig 6B shows the corresponding spectral density. Thus, parameter values presented in S1 Table and S2 Table result in low frequency oscillations (delta, theta and alpha) with highest power in layers 5 and 6 while gamma oscillations have its main power in layer 2/3. This is in agreement with recent findings suggesting that gamma activity is predominant in superficial layers while lower frequencies are predominant in deep layers [70, 71]. We then proceeded to test the existence of PAC. For this, we filtered each time series in Fig 4 into five frequency bands from delta to gamma and applied the Hilbert transform to obtain instantaneous phases and amplitudes for each frequency band and each neuronal population.

**Fig 7:**
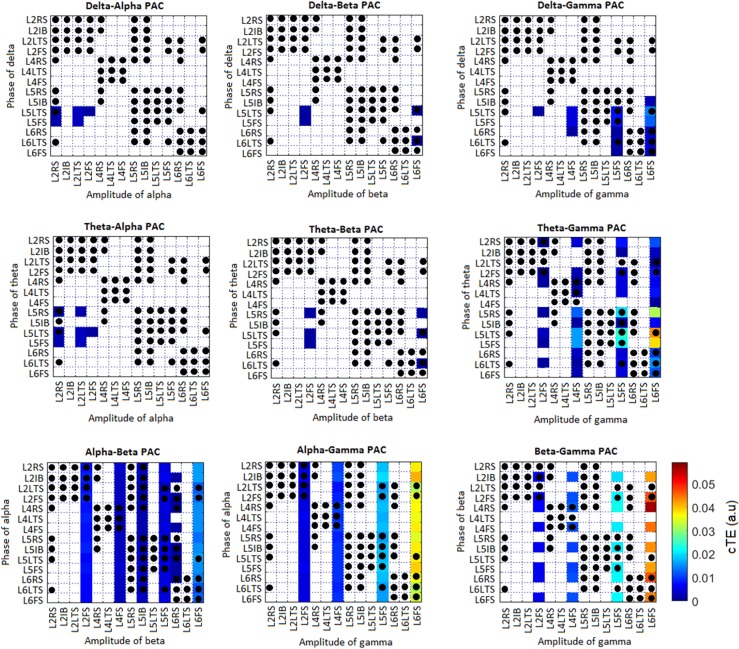
Phase-amplitude coupling (PAC) corresponding to the simulation presented in Fig 4. Non-significant values were set to zero and are depicted in white. Black dots indicate existing anatomical connections (see Fig 1).

Ten different PAC combinations between a low-frequency phase and a higher-frequency amplitude were computed using the cTE measure: delta-theta, delta-alpha, delta-beta, delta-gamma, theta-alpha, theta-beta, theta-gamma, alpha-beta, alpha-gamma, and beta-gamma. Each PAC combination consisted of a matrix of 14x14 cTE values representing all possible interactions between the 14 neuronal populations. To test the significance of these values, surrogate data was computed, followed by a multiple comparison analysis (see Methods). Results include nine out of the ten PAC combinations (Fig 7). The delta-theta PAC combination was not included since no significant values were obtained for the set of parameters used.

The strongest PAC value found was between the phase of the beta band in L4RS and the amplitude of the gamma band in L6FS, which we will denote as L4RS→ L6FS. In fact, the strongest values found involved the amplitude of gamma in L6. For example, additional strong connections in the beta-gamma combination were L2FS→L6FS, L5FS→L6FS, L4FS→L6FS, L6RS→L6FS, and L2IB→L6FS. Alpha-gamma (L2IB→L6FS, L5FS→L6FS) and theta-gamma (L5LTS→L6FS, L5FS→L6FS) combinations also presented strong connections. Some of these values do not represent direct connections between the populations. For example, the strongest connection, beta-gamma (L4RS→ L6FS), does not correspond to an anatomical (direct) connection (see Fig 1). Thus, we emphasize that PAC matrices (Fig 7) represent effective connections, which may not correspond to anatomical connections. To make this clearer, anatomical connections in Fig 7 are represented with black dots.

### Parameter sensitivity analysis

Some of the parameters presented in S1 Table and S2 Table were taken from the literature [8, 31, 72], and parameters with no equivalent in the literature were assigned physiologically reasonable values. Thus, it is necessary to explore how changes in these parameters can affect PAC values. In this section, for the sake of simplicity, we focus on three PAC combinations which involve the gamma rhythm and have been of great interest in the literature: delta-gamma, theta-gamma, and alpha-gamma.

### Controlling the strength of PAC

We selected nine different parameters and explored how their change affected the strength of the PAC phenomenon. For each parameter we considered 100 different values and thus performed 100 different simulations. The parameters were: 1) a multiplying factor *η* = 0.03:0.03:3 controlling the global strength of the connectivity matrix (Γ_*nm*_ = *η*Γ_*nm*_), 2) the reciprocal of the time constant of RS populations (*k*_*RS*_ = 5:5:500*s*^−1^), 3) the reciprocal of the time constant of IB populations (*k*_*IB*_ = 5:5:500*s*^−1^), 4) the reciprocal of the time constant of LTS populations (*k*_*LTS*_ = 5:5:500*s*^−1^), 5) the reciprocal of the time constant of FS populations (*k*_*FS*_ = 5:5:500*s*^−1^), 6) the external input to the L4RS population (${\bar{p}}_{5}=10:10:1000\;s^{-1},\sigma_{p_{5}}=1s^{-1}$), 7) the external input to the L4FS population (${\bar{p}}_{7}=10:10:1000\;s^{-1},\sigma_{p_{7}}=1\;s^{-1}$), 8) the gains of the six excitatory populations (*G*_*E*_ ≡ *G*_1_ = *G*_2_ = *G*_5_ = *G*_8_ = *G*_9_ = *G*_12_ = 0.2:0.2:20*mV*), and 9) the gains of the eight inhibitory populations (*G*_*I*_ ≡ *G*_3_ = *G*_4_ = *G*_6_ = *G*_7_ = *G*_10_ = *G*_11_ = *G*_13_ = *G*_14_ = 0.5:0.5:50*mV*).

**Fig 8:**
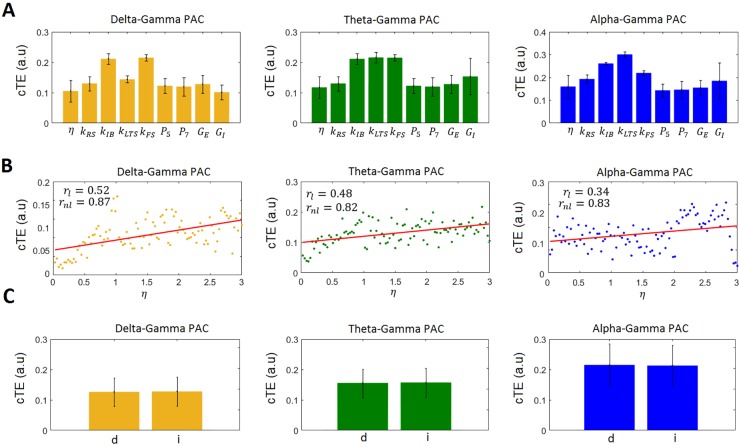
Exploring the parameter space for three different PAC combinations. A) Average cTE values for delta-gamma (orange), theta-gamma (green), and alpha-gamma (blue) PAC when considered 100 different values for nine different parameters: 1) a multiplying factor *η* = 0.03:0.03:3 controlling the global strength of the connectivity matrix (Γ_*nm*_ = *η*Γ_*nm*_), 2) the reciprocal of the time constant of RS populations (*k*_*RS*_ = 5:5:500*s*^−1^), 3) the reciprocal of the time constant of IB populations (*k*_*IB*_ = 5:5:500*s*^−1^), 4) the reciprocal of the time constant of LTS populations (*k*_*LTS*_ = 5:5:500*s*^−1^), 5) the reciprocal of the time constant of FS populations (*k*_*FS*_ = 5:5:500*s*^−1^), 6) the external input to the L4RS population (${\bar{p}}_{5}=10:10:1000\;s^{-1},\sigma_{p_{5}}=1s^{-1}$), 7) the external input to the L4FS population (${\bar{p}}_{7}=10:10:1000\;s^{-1},\sigma_{p_{7}}=1s^{-1}$), 8) the gains of the six excitatory populations (*G*_*E*_ ≡ *G*_1_ = *G*_2_ = *G*_5_ = *G*_8_ = *G*_9_ = *G*_12_ = 0.2:0.2:20*mV*), and 9) the gains of the eight inhibitory populations (*G*_*I*_ ≡ *G*_3_ = *G*_4_ = *G*_6_ = *G*_7_ = *G*_10_ = *G*_11_ = *G*_13_ = *G*_14_ = 0.5:0.5:50*mV*). B) Plot of cTE versus *η*. C) Average cTE values for direct and indirect PAC connections. Labels ‘d’, and ‘i’ correspond to direct and indirect PAC connections.

Then, for each PAC combination we obtained 14x14x100 = 19600 cTE values (although many of them are zero). We summarized that information by taking the strongest value found in each simulation, which results in a series of 100 values for each PAC combination. Fig 8A displays the mean and standard deviation of the 100-point series of the strongest PAC values for the three PAC combinations considered. In the figure, delta-gamma PAC is depicted in orange, theta-gamma PAC in green, and alpha-gamma PAC in blue. Our results shows that for the three PAC combinations, the highest increases in cTE are obtained when changing the reciprocal time constants of the populations. This result is not surprising since these parameter control the frequency at which the populations oscillate.

The exploration of the parameter space is important because PAC has been suggested to be the carrier mechanism for the interaction of local and global processes in the brain, and is thus directly related to the integration of distributed information in the brain [1]. Neuronal circuits can thus control the amount of information transmitted in the PAC phenomenon by changing the values of physiological parameters of specific populations.

### On the influence of the connectivity matrix Γ_*nm*_ on PAC strength

An important problem in neuroscience is the link between structural and functional brain networks [73, 74]. In the context of this work, it is of interest to study the influence of the connectivity matrix Γ_*nm*_ on the generated PAC phenomenon.

Fig 8B displays the series of 100 PAC values versus the factor *η*. The solid line corresponds to the fit of a linear model. The linear correlation values between delta-gamma, theta-gamma, alpha-gamma and *η* were 0.52, 0.48, and 0.34, respectively. We then performed a nonlinear regression analysis (Eq 18) with *η* as the regressor and computed the nonlinear correlation coefficient. The nonlinear correlation values between delta-gamma, theta-gamma, alpha-gamma and *η* were 0.87, 0.82, and 0.83, respectively, showing that there is a strong nonlinear relationship between the strength of PAC and effective connectivity between the populations involved. The values were significant as tested with the surrogate data approach.

We also counted all significant PAC connections obtained in the 100 simulations. The vectors of significant connections for delta-gamma, theta-gamma, and alpha-gamma PAC comprised 800, 1998, and 2000, cTE values, respectively, for the PAC interactions that have a corresponding anatomical connection (direct interactions), and 1595, 3593, and 3600 for the interactions without an anatomical equivalent (indirect interactions). The mean and standard deviations of these connections are presented in Fig 8C. Our results showed that for the three PAC combinations, there was not a statistically significant difference between the average strength of direct and indirect PAC interactions (as tested with a t-test, p<0.05).

**Fig 9:**
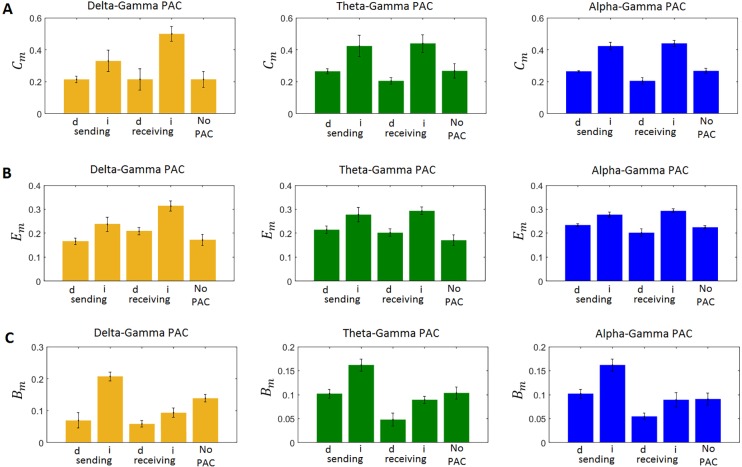
The link between local topological measures and PAC. A) local clustering coefficient (*C*_*m*_), B) local efficiency (*E*_*m*_), C) local betweenness centrality (*B*_*m*_). In all panels, labels ‘d’, and ‘i’ correspond to direct and indirect PAC connections, respectively. Populations can send and/or receive PAC interactions, or they can be not involved in the generation of PAC.

**Fig 10:**
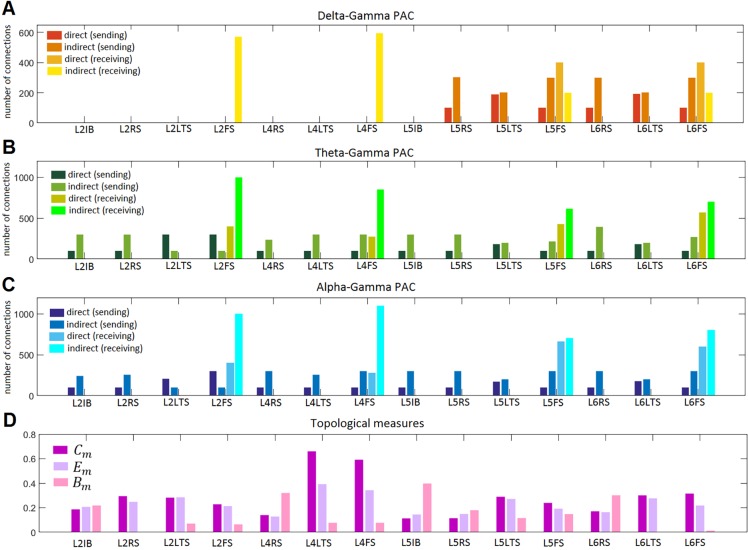
Population statistics. A) number of delta-gamma PAC connections, B) number of theta-gamma PAC connections, C) number of alpha-gamma PAC connections, D) topological measures: local clustering coefficient (*C*_*m*_), local efficiency (*E*_*m*_), and local betweenness centrality (*B*_*m*_). In all panels, connections can be direct or indirect, and populations can send and/or receive PAC interactions, or they can be not involved in the generation of PAC.

We also computed three local topological measures for the network of 14 coupled neuronal populations (Fig 1A): *C*_*m*_, *E*_*m*_, and *B*_*m*_. The edges of the network were the absolute values of the connections between the populations (Fig 1B). We found that on average, indirect PAC interactions are made by populations with higher *C*_*m*_ (Fig 9A), *E*_*m*_ (Fig 9B), and *B*_*m*_ (Fig 9C) than populations making direct connections, and populations not involved in PAC connections. Populations receiving indirect PAC connections had also on average higher topological measures than populations receiving direct interactions. This can be also appreciated in Fig 10, where we plotted the number of PAC connections sent and received by each population (Fig 10A, 10B and 10C) as well as the topological properties of the populations (Fig 10D). In the case of the delta-gamma combination, the populations sending the highest number of direct connections were L6LTS, and L5LTS, and the populations sending the highest number of indirect connections were L5FS, L6FS, L5RS, and L6RS. On the other hand, the populations receiving the highest number of direct connections were L5FS and L6FS, whereas the populations receiving the highest number of indirect connections were L4FS and L2FS. From Fig 10A we see that for all populations with a statistically significant number of connections the number of indirect connections that were sent was higher than the number of direct connections that were sent. In the case of received connections, superficial populations (L2FS and L4FS) received more indirect than direct connections, whereas deeper populations (L5FS and L6FS) received more direct connections than indirect connections. However, when counting the entire cortical column, more indirect connections were received than direct connections. Superficial populations L2FS and L4FS also received the highest number of indirect connections when considering the theta-gamma and alpha-gamma combinations (Fig 10B and 10C). Deep populations L5FS and L6FS received the highest number of direct connections. Superficial populations L2LTS and L2FS sent the highest number of direct connections while deep population L6RS sent the highest number of indirect connections. Fig 10D shows the three topological measures for each population. L4LTS and L4FS presented the highest clustering coefficient and efficiency, whereas L5IB, L4RS, and L6RS presented the highest betweenness centrality. When taking into account all populations and all connections (Fig 10A, 10B and 10C) the result presented in Fig 9 is obtained: indirect connections presented higher value of topological measures than direct connections.

### Generation of other types of CFC

The neural mass model presented in this paper can generate rich temporal dynamics. Studies of the dynamics generated by the Jansen and Rit model, which is the basis for our model, can be found elsewhere [46, 75, 76]. In this paper we focused on PAC, but this is only one type of the general phenomenon of CFC which results from nonlinearities in brain dynamics. It is thus not unexpected to find other types of CFC in the signals generated by our model (for example, the temporal dynamics of L5RS in Fig 4 presents frequency modulation). In addition to PAC, other types of CFC such as AAC, PPC, and phase-frequency coupling (PFC) have been explored in the literature [77, 78] and could all be calculated using Eq (8) after replacing *a*_*nl*_(*t*) and *φ*_*mk*_(*t*) with the appropriate time series.

**Fig 11:**
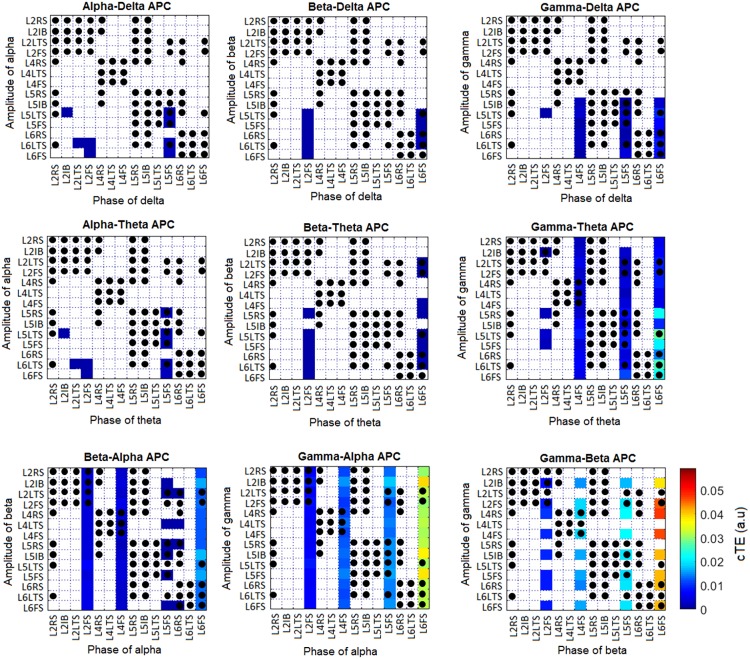
Amplitude-phase coupling (APC) corresponding to the simulation presented in Figs 4 and 5. Non-significant values were set to zero and are depicted in white. Black dots indicate existing anatomical connections (see Fig 1).

Recently, the analysis of resting state electrocorticography (ECoG) data revealed that the amplitude of gamma oscillations can drive the phase of alpha oscillations, i.e, APC [33]. Although this experimental result may seem surprising, it is consistent with theoretical results in the NMM literature. Specifically, starting with a network of weakly coupled Wilson and Cowan oscillators, equations for the instantaneous phases were obtained which depended on the instantaneous amplitudes of the oscillators in the network [55]. Thus, by setting different natural frequencies for the oscillators in the network, it is possible to obtain not only PAC but other types of CFC. To test the existence of APC we computed:

$$cTE_{a_{nl}\to \varphi_{mk}}=\frac{1}{N}\sum_{\delta =1}^{N}cMI\left(a_{nl},\varphi_{mk}^{\delta }|\tilde{M}\right)$$ (24)

where $\varphi_{mk}^{\delta }$ is derived from the phase time series *φ*_*mk*_ at *δ* steps into the future, i.e. $\varphi_{mk}^{\delta }=\varphi_{mk}\left(t+\delta \right)$, and $\tilde{M}$ is a matrix comprising all phases and amplitudes in all populations, except *a*_*nl*_. Fig 11 shows the APC estimated from the simulated data presented in Figs 4 and 5, with the strongest values corresponding to the gamma-beta and gamma-alpha APC combinations. Thus, our simulations are in agreement with recent experimental evidence suggesting the existence of APC [33].

### Mechanisms mediating indirect PAC interactions

**Fig 12:**
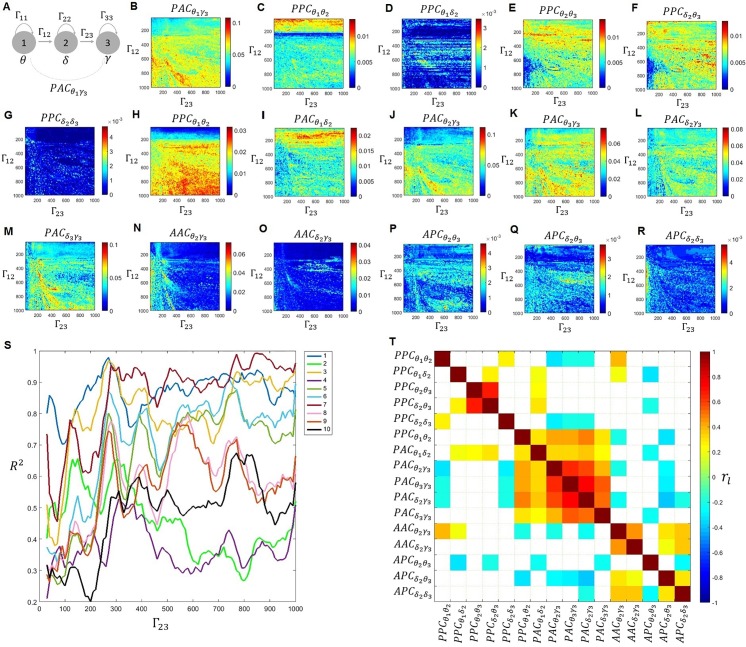
Mechanisms mediating indirect PAC connections (Case I). A) Three population toy model comprising three neuronal populations labelled as ‘1’, ‘2’, and ‘3’, oscillating in the theta (*θ*), delta (*δ*), and gamma (*γ*) bands. B) PAC involving the phase of theta in population 1 and the amplitude of gamma in population 3 ($PAC_{\theta_{1}\gamma_{3}}$) obtained when varying the connectivity parameters between populations 1 and 2 (Γ_12_ = 30:4:1000) and between populations 2 and 3 (Γ_23_ = 30:4:1000). Panels C to R, displays the 16 predictors used in the ten models explored (Table 2). S) Coefficient of determination (*R*^2^) for the ten models explored (Table 2). T) Correlation coefficient between the 16 predictors.

**Fig 13:**
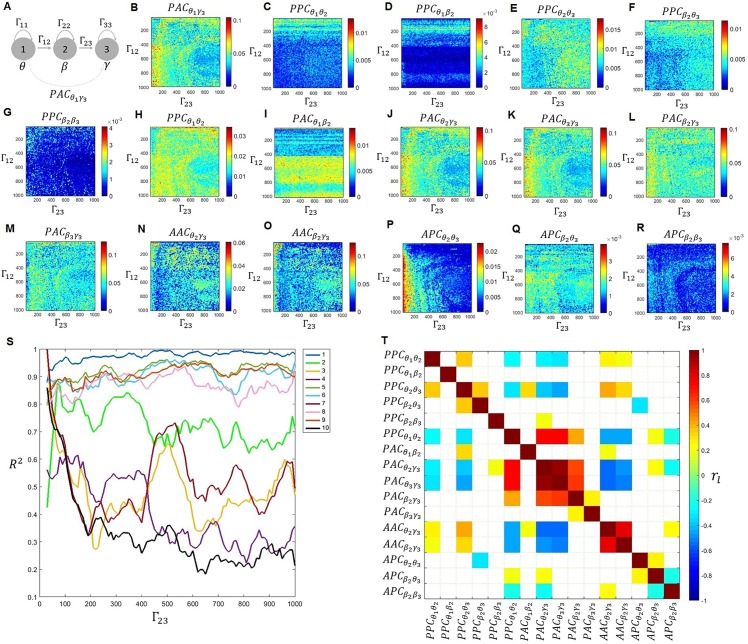
Mechanisms mediating indirect PAC connections (Case II). A) Three population toy model comprising three neuronal populations labelled as ‘1’, ‘2’, and ‘3’, oscillating in the theta (*θ*), beta (*β*), and gamma (*γ*) bands. B) PAC involving the phase of theta in population 1 and the amplitude of gamma in population 3 ($PAC_{\theta_{1}\gamma_{3}}$) obtained when varying the connectivity parameters between populations 1 and 2 (Γ_12_ = 30:4:1000) and between populations 2 and 3 (Γ_23_ = 30:4:1000). Panels C to R, display the 16 predictors used in the ten models explored (Table 2). S) Coefficient of determination (R^2^) for the ten models explored (Table 2). T) Correlation coefficient between the 16 predictors.

We demonstrated theoretically (see Methods), that given three neuronal populations connected in such a way that there is only an indirect connection between populations 1 and 3:1→2→3, the indirect PAC connection from population 1 to population 3 $\left(cTE_{\varphi_{1}⇝a_{3}}\right)$ can be computed as the sum of the direct connections $cTE_{\varphi_{1}\to y_{2}}$ and $cTE_{y_{2}\to a_{3}}$, plus a term $\tilde{I}$ comprising a sum of mutual information terms (see Eqs 16 and 17). $cTE_{\varphi_{1}\to y_{2}}$ and $cTE_{y_{2}\to a_{3}}$ can have the interpretation of CFC or SFC, depending on the interpretation given to *y*_2_. Given the complicated mathematical expression for $\tilde{I}$ (Eq 17), it is tempting to write Eq (16) for $cTE_{\varphi_{1}⇝a_{3}}$ in terms of only transfer entropy terms (i.e, CFC and SFC variables). Since such a closed mathematical expression for $cTE_{\varphi_{1}⇝a_{3}}$ does not seems to exists, we explore here different approximations (models in Table 2) via numerical simulations (Figs 12 and 13).

**Table 2:** Indirect PAC modeled as a cascade of direct CFC and SFC in a three population network. Two cases were considered: population 2 oscillates in the delta (*δ*) band (Case I), and population 2 oscillates in the beta (*β*) band (Case II). Populations 1 and 3 always oscillate in the theta (*θ*) and gamma (*γ*) bands, respectively.

Model	Case I	Case II
1	$PAC_{\theta_{1}\gamma_{3}}=PPC_{\theta_{1}\theta_{2}}+PAC_{\theta_{2}\gamma_{3}}$	$PAC_{\theta_{1}\gamma_{3}}=PPC_{\theta_{1}\theta_{2}}+PAC_{\theta_{2}\gamma_{3}}$
2	$PAC_{\theta_{1}\gamma_{3}}=PAC_{\theta_{1}\theta_{2}}+AAC_{\theta_{2}\gamma_{3}}$	$PAC_{\theta_{1}\gamma_{3}}=PAC_{\theta_{1}\theta_{2}}+AAC_{\theta_{2}\gamma_{3}}$
3	$PAC_{\theta_{1}\gamma_{3}}=PPC_{\theta_{1}\delta_{2}}+PAC_{\delta_{2}\gamma_{3}}$	$PAC_{\theta_{1}\gamma_{3}}=PPC_{\theta_{1}\beta_{2}}+PAC_{\beta_{2}\gamma_{3}}$
4	$PAC_{\theta_{1}\gamma_{3}}=PAC_{\theta_{1}\delta_{2}}+AAC_{\delta_{2}\gamma_{3}}$	$PAC_{\theta_{1}\gamma_{3}}=PAC_{\theta_{1}\beta_{2}}+AAC_{\beta_{2}\gamma_{3}}$
5	$PAC_{\theta_{1}\gamma_{3}}=PPC_{\theta_{1}\theta_{2}}+PPC_{\theta_{2}\theta_{3}}+PAC_{\theta_{3}\gamma_{3}}$	$PAC_{\theta_{1}\gamma_{3}}=PPC_{\theta_{1}\theta_{2}}+PPC_{\theta_{2}\theta_{3}}+PAC_{\theta_{3}\gamma_{3}}$
6	$PAC_{\theta_{1}\gamma_{3}}=PPC_{\theta_{1}\delta_{2}}+PPC_{\delta_{2}\theta_{3}}+PAC_{\theta_{3}\gamma_{3}}$	$PAC_{\theta_{1}\gamma_{3}}=PPC_{\theta_{1}\beta_{2}}+PPC_{\beta_{2}\theta_{3}}+PAC_{\theta_{3}\gamma_{3}}$
7	$PAC_{\theta_{1}\gamma_{3}}=PPC_{\theta_{1}\delta_{2}}+PPC_{\delta_{2}\delta_{3}}+PAC_{\delta_{3}\gamma_{3}}$	$PAC_{\theta_{1}\gamma_{3}}=PPC_{\theta_{1}\beta_{2}}+PPC_{\beta_{2}\beta_{3}}+PAC_{\beta_{3}\gamma_{3}}$
8	$PAC_{\theta_{1}\gamma_{3}}=PAC_{\theta_{1}\theta_{2}}+APC_{\theta_{2}\theta_{3}}+PAC_{\theta_{3}\gamma_{3}}$	$PAC_{\theta_{1}\gamma_{3}}=PAC_{\theta_{1}\theta_{2}}+APC_{\theta_{2}\theta_{3}}+PAC_{\theta_{3}\gamma_{3}}$
9	$PAC_{\theta_{1}\gamma_{3}}=PAC_{\theta_{1}\delta_{2}}+APC_{\delta_{2}\theta_{3}}+PAC_{\theta_{3}\gamma_{3}}$	$PAC_{\theta_{1}\gamma_{3}}=PAC_{\theta_{1}\beta_{2}}+APC_{\beta_{2}\theta_{3}}+PAC_{\theta_{3}\gamma_{3}}$
10	$PAC_{\theta_{1}\gamma_{3}}=PAC_{\theta_{1}\delta_{2}}+APC_{\delta_{2}\delta_{3}}+PAC_{\delta_{3}\gamma_{3}}$	$PAC_{\theta_{1}\gamma_{3}}=PAC_{\theta_{1}\beta_{2}}+APC_{\beta_{2}\beta_{3}}+PAC_{\beta_{3}\gamma_{3}}$

For the simulations, we set population 1 to oscillate in the theta band while population 3 oscillated in the gamma band. Two different cases were considered for population 2. Case I (Fig 12): population 2 oscillated with a frequency lower (delta band) than population 1, and case II (Fig 13): population 2 oscillated with a frequency higher (beta band) than population 1. Five different types of CFC between the three populations (PAC, APC, PPC, AAC, and PFC) were estimated while varying the connectivity parameters between populations 1 and 2 (Γ_12_) and between populations 2 and 3 (Γ_23_). SFC was also considered and labelled in the same way as the CFC interactions. For example, $PPC_{\theta_{1}\theta_{2}}$, is the cTE from the phase of theta in population 1 to the phase of theta in population 2. To test the significance of these values, surrogate data was computed, followed by a multiple comparison analysis. As a control, we computed the cTE from populations 2 and 3 to population 1 for all possible types of interactions (such as $PPC_{\theta_{2}\theta_{1}}$), and confirmed they were not statistically significant.

There are several pathways that could transfer information from the phase of the theta oscillation in population 1 to the amplitude of the gamma oscillation in population 3 ($PAC_{\theta_{1}\gamma_{3}}\equiv cTE_{\varphi_{1}⇝a_{3}}$). For example, a simple model could involve $PPC_{\theta_{1}\theta_{2}}$ followed by PAC between the theta rhythm in population 2 and the gamma rhythm in population 3: ${PAC}_{\theta_{1}\gamma_{3}}={PPC}_{\theta_{1}\theta_{2}}+{PAC}_{\theta_{2}\gamma_{3}}$. A more complicated one is: $PAC_{\theta_{1}\gamma_{3}}=PPC_{\theta_{1}\delta_{2}}+PPC_{\delta_{2}\delta_{3}}+PAC_{\delta_{3}\gamma_{3}}$. To compare different models for $PAC_{\theta_{1}\gamma_{3}}$ (Table 2), we fitted a linear regression to $PAC_{\theta_{1}\gamma_{3}}$ and computed the coefficient of determination (*R*^*2*^) as a function of parameter Γ_23_. Note that models 1 to 4 in Table 2 correspond to $\tilde{I}\approx 0$.

For Case I we found that the three best models were Model 1 ($PAC_{\theta_{1}\gamma_{3}}=PPC_{\theta_{1}\theta_{2}}+PAC_{\theta_{2}\gamma_{3}}$), Model 7 ($PAC_{\theta_{1}\gamma_{3}}=PPC_{\theta_{1}\delta_{2}}+PPC_{\delta_{2}\delta_{3}}+PAC_{\delta_{3}\gamma_{3}}$), and Model 3 ($PAC_{\theta_{1}\gamma_{3}}=PPC_{\theta_{1}\delta_{2}}+PAC_{\delta_{2}\gamma_{3}}$), with Model 1 being the best model for low values of Γ_23_, and Model 7 for the high values. On the other hand, we obtained the opposite behavior for Case II, i.e, Model 7 was the best model for low Γ_23_ values, whereas Model 1 was better at explaining $PAC_{\theta_{1}\gamma_{3}}$ for high Γ_23_ values. The correlation between the 16 CFC and SFC variables involved in the ten models is displayed in the last panel for both cases (Figs 12 and 13). Although we found significant PFC combinations, models involving these combinations were very weak predictors of $PAC_{\theta_{1}\gamma_{3}}$ (with R^2^ <0.08 in all cases) and were thus not included in Table 2 and Figs 12 and 13.

## Discussion

We have analyzed a neural mass model that captures the phase-amplitude coupling between layers in a cortical column. The model comprises fourteen interconnected neuronal populations distributed across four cortical layers (L2/3, L4, L5 and L6). According to our results, the parameters with the strongest influence on the strength of PAC were the time constants.

As expected, in order to generate PAC, nonlinearities in the model are essential. As was shown in Fig 2E, when the sigmoid function was substituted with a linear function, no modulation was obtained. Additionally, the strength of PAC was best modeled by a nonlinear regression of the connectivity values instead of a linear regression. Thus, our results show that the nonlinear interaction of neuronal populations (via the sigmoid function and the connectivity matrix) can produce PAC combinations with frequencies different from the natural frequencies of the oscillators involved. Our model of oscillators with natural frequencies in the theta, alpha and gamma bands was able to produce significant PAC involving other bands such as delta and beta: delta-alpha, delta-beta, delta-gamma, theta-beta, alpha-beta, and beta-gamma. Interestingly, some peaks in the beta band are harmonics of theta and alpha oscillations, such as the beta peak at 16.83 Hz in the spectrum of L4FS in Fig 5. Due to the interaction between the populations, there is a statistically significant PAC from the phase of beta in L4FS to the amplitude of gamma in L2FS, L4FS, L5FS and L6FS. Note that of these PAC interactions, only L4FS → L4FS corresponds to an anatomical connection (Fig 1B and S2 Table). If we take into account all PAC combinations in Fig 7, less than 40% of all significant PAC values (93/238 = 39.08%) corresponded to anatomical connections. This suggests that although effective connections are constrained by direct (anatomical) connections additional factors are needed to fully explain the link between anatomical and effective connectivity. Interestingly, our numerical simulations showed that on average the strength of the PAC phenomenon mediated by direct and indirect connections is approximately the same (Fig 8). However, local topological measures such as clustering coefficient, efficiency, and betweenness centrality were the highest for populations making indirect connections when compared to populations making direct PAC connections, to populations receiving PAC connections, and to populations not involved on the generation of PAC. This is another factor to consider when studying the origin of PAC during neurodegenerative disorders known to affect both local and global brain circuitry [79–81].

One limitation of the present approach is that model parameters are loosely constrained from existing neurophysiological data. Thus, although our model provides insight about the emergence of PAC in a complex network whose spectral and connectivity properties resemble that of a cortical column, specific conclusions should await to more knowledge of these data.

### Comparison with previous models of PAC generation

The first computational models of PAC generation were realistic models of the theta-gamma coupling in the hippocampus [3]. These models considered networks of hundreds of interconnected neurons which were individually modeled by either a single compartment [4] or realistically represented by multiple compartments for the soma, axon, and dendrites [5]. A practical disadvantage of this approach is that it needs high computational power, but more importantly, the use of such realistic models produces hundreds or thousands of variables and parameters, making it difficult to determine their influence on the generated average network characteristics. This is especially critical if we are interested in analyzing the link of PAC and mesoscopic phenomena like functional magnetic resonance signals [23]. The analysis of multiple PAC combinations as done in this paper would be even more difficult with realistic networks. By comparison, our model of one cortical column comprised only 14 second-order (or 28 first-order) differential equations, which can be easily solved using any modern personal computer.

Additionally, previous models of PAC generation, both the ones based on realistic networks [3] and the ones based on neural mass models [6] studied the phenomenon in a qualitative way, such that they did not actually compute a PAC measure but limited their analysis to the generation of temporal dynamics resembling PAC. This makes it difficult to compare their results with our quantitative approach based on information theory.

### Indirect PAC connections can be predicted by a cascade of direct CFC and interactions within the same frequency band

As a unifying theory of EEG organization, it has been proposed that the EEG is hierarchically organized such that the delta phase modulates the theta amplitude, and the theta phase modulates the gamma amplitude [10]. It was also proposed that this oscillatory hierarchy controls baseline excitability and that the hierarchical organization of ambient oscillatory activity allows the auditory cortex to structure its temporal activity pattern to optimize the processing of rhythmic inputs. Recent findings suggest a somewhat different hierarchy of oscillatory activity with regard to these frequency bands [22]. Sotero et al. did not observe PAC between the delta and theta bands in rat area S1FL: PAC was statistically significant between the phases of the delta and theta bands and the amplitudes of the beta and gamma bands, but not between the phase of the delta band and the amplitude of the theta band. Their data support specific PAC interactions, but not a clear hierarchical PAC structure. The differences between Lakatos et al.’s findings and Sotero et al.’s findings are consistent with their proposal that the hierarchical structure found in the auditory cortex may support processing of rhythmic auditory stimuli, which are less common in natural somatosensory stimuli to the forepaw. Both studies were restricted to PAC and did not explore whether the oscillatory hierarchy might involve other types of CFC. While historically PAC and PPC have been the subject of most experimental and modeling studies, other types of CFC are attracting increasing interest [33, 77, 78]. Our theoretical and numerical results show that indirect PAC is better understood if analyzed together with direct PAC and other types of direct CFC and SFC connections. Our results do not suggest a specific oscillatory hierarchy like the one proposed by Lakatos et al., but multiple contributing cascades of CFC and SFC. Future analysis of experimental data will need to determine the functional importance of these different possible pathways.

### cTE as a unified approach to estimate CFC

In this work, we used the average cTE, computed using the conditional mutual information [56, 57] to measure the influence of the phase of a low frequency rhythm on the amplitude of a higher frequency rhythm, and used it as an index of PAC. A known limitation of the cTE approach is that it requires long time series [60]. For this reason, we used time series comprising of up to 10^5^ time instants. Recent studies have shown that cTE is biased as its values depend on the autodependency coefficient in a dynamical system [82]. Conditional TE was chosen over pairwise mutual information [53] or the pairwise information flow [83] because pairwise analysis cannot distinguish between connectivity configurations such as [X→Y, X→Z, Z→Y] and [X→Z, Z→Y] [84].

An advantage of measures based on information theory is that they are model-free. This is in contrast to other measures like Granger causality, which are based on autoregressive models [85]. Furthermore, Granger causality should not be applied to band-passed signals because the filtering process produces a large increase in the empirical model order, which often results in spurious results [86]. Another advantage of the cTE measure is that it can be used to estimate any type of CFC, not just PAC. Thus, it provides a unified measure to study the CFC phenomenon.

cTE has often been given a causal interpretation, however a more recent point of view [87] suggests that cTE should be interpreted as predictive information transfer, i.e. the amount of information that a source variable adds to the next state of the destination variable. Ultimately, interventions are required to detect causal interactions [88]. This formalism is used in a causal measure called information flow [89], which is also based on the cMI.

## Supporting Information

S1 Table Values and physiological interpretations of model parameters for the 14 neuronal populations. (DOCX)

S2 Table Standard values of the anatomically constrained effective connectivity matrix Γ_*nm*_ (Fig 1B). All values represent anatomical (direct) connections. Values that are zero were taken from the literature [31]. Nonzero values were also taken from [31] and some of them were manually tuned to produce peaks in the spectrum of *x*_*m*_(*t*) in all frequencies of interest (from delta to gamma) as well as an average LFP spectrum (Fig 6) consistent with experimental results [69, 71].
(DOCX)

S3 Table Values of model parameters for the three-population model (Fig 2). (DOCX)
