# Supplementary material for: Topology, Cross-Frequency, and Same-Frequency Band Interactions Shape the Generation of Phase-Amplitude Coupling in a Neural Mass Model of a Cortical Column
Source: PLoS Comput Biol. 2016 Nov 1;12(11):e1005180. doi: 10.1371/journal.pcbi.1005180 (PMC5089773; doi:10.1371/journal.pcbi.1005180)
Supplement: S1 Table — (DOCX) [file pcbi.1005180.s001.docx]

| **Parameter [units]** | **Interpretation** | **Value** | **Reference** |
| --- | --- | --- | --- |
| $G\left[ mV \right]$ | Gain | $G_{1}= 5.17$ $G_{2}= 5.17$  $G_{3}= 4.45$ $G_{4}= 50.00$  $G_{5}= 5.17$ $G_{6}= 4.45$  $G_{7}=40.00$ $G_{8}= 5.17$ $G_{9}= 5.17$ $G_{10}= 4.45$ $G_{11}=40.00$ $G_{12}= 5.17$ $G_{13}= 4.45$ $G_{14}=30.00$ | ([Wendling, Bellanger et al. 2000](#_ENREF_87), [Zavaglia, Astolfi et al. 2006](#_ENREF_91)) |
| $g\left[ s^{-1} \right]$ | Reciprocal of time constant | $g_{1}= 60$ $g_{2}= 70$  $g_{3}= 30$ $g_{4}=300$  $g_{5}= 60$ $g_{6} = 30$  $g_{7}=250$ $g_{8}= 60$  $g_{9}= 70$ $g_{10}= 30$  $g_{11}=250$ $g_{12}= 60$  $g_{13}= 30$ $g_{14}=250$ | ([Wendling, Bellanger et al. 2000](#_ENREF_87), [Zavaglia, Astolfi et al. 2006](#_ENREF_91)) |
| $\bar{p}\left[ s^{-1} \right]$,$\sigma_{p}\left[ s^{-1} \right]$ | Mean and standard deviation of the external Gaussian input | $\bar{p}_{i}=0$ for $i\neq\left\{ 5,7 \right\}$ ,$\bar{p}_{5}=10$, $\bar{p}_{7}=5$  $\sigma_{p_{i}}=0$for $i\neq\left\{ 5,7 \right\}$ , $\sigma_{p_{5}}=0$, $\sigma_{p_{7}}=0$ | - |
| $b$ | Damping coefficient | $b=0.001$ for all populations | - |
| $e_{0}\left[ s^{-1} \right]$ | Maximum firing rate | $e_{0}=5$ for all populations | ([Jansen and Rit 1995](#_ENREF_34)) |
| $v_{0}\left[ mV \right]$ | Position of the sigmoid function | $v_{0}=6$ for all populations | ([Jansen and Rit 1995](#_ENREF_34)) |
| $r\left[ mV^{-1} \right]$ | Steepness of the sigmoid function | $r=0.56$ for all populations | ([Jansen and Rit 1995](#_ENREF_34)) |
