# Supplementary material for: Topology, Cross-Frequency, and Same-Frequency Band Interactions Shape the Generation of Phase-Amplitude Coupling in a Neural Mass Model of a Cortical Column
Source: PLoS Comput Biol. 2016 Nov 1;12(11):e1005180. doi: 10.1371/journal.pcbi.1005180 (PMC5089773; doi:10.1371/journal.pcbi.1005180)
Supplement: S2 Table — All values represent anatomical (direct) connections. Values that are zero were taken from the literature [31]. Nonzero values were also taken from [31] and some of them were manually tuned to produce peaks in the spectrum of xm(t) in all frequencies of interest (from delta to gamma) as well as an average LFP spectrum (Fig 6) consistent with experimental results [69, 71]. (DOCX) [file pcbi.1005180.s002.docx]

| From To | | **L2/3** | | | | **L4** | | | **L5** | | | | **L6** | | |
| --- | --- | --- | --- | --- | --- | --- | --- | --- | --- | --- | --- | --- | --- | --- | --- |
|  |  | **RS** | **IB** | **LTS** | **FS** | **RS** | **LTS** | **FS** | **RS** | **IB** | **LTS** | **FS** | **RS** | **LTS** | **FS** |
| **L2/3** | **RS** | 19.23 | 12.53 | 34.17 | 14.07 | 1.61 | 0 | 0 | 3.82 | 1.61 | 0 | 0 | 0 | 0 | 0 |
|  | **IB** | 12.53 | 12.53 | 27.47 | 14.07 | 1.61 | 0 | 0 | 3.82 | 1.61 | 0 | 0 | 0 | 0 | 0 |
|  | **LTS** | -23.45 | -23.45 | -52.93 | -6.70 | 0 | 0 | 0 | -23.45 | -33.50 | 0 | -16.75 | -16.75 | 0 | -11.39 |
|  | **FS** | -3.35 | -5.36 | -6.03 | -20.1 | 0 | 0 | 0 | -3.35 | -6.70 | 0 | -2.01 | -3.35 | 0 | -2.01 |
| **L4** | **RS** | 9.72 | 0 | 0 | 0 | 22.98 | 34.17 | 58.96 | 7.77 | 8.17 | 0 | 0 | 2.14 | 0 | 0 |
|  | **LTS** | 0 | 0 | 0 | 0 | -23.45 | -52.93 | -8.71 | 0 | 0 | 0 | 0 | 0 | 0 | 0 |
|  | **FS** | 0 | 0 | 0 | 0 | -6.03 | -6.03 | -61.64 | 0 | 0 | 0 | 0 | 0 | 0 | 0 |
| **L5** | **RS** | 1.47 | 0 | 0 | 0 | 0.47 | 0 | 0 | 32.89 | 5.36 | 20.77 | 8.71 | 2.14 | 0 | 0 |
|  | **IB** | 1.21 | 0 | 0 | 0 | 0.47 | 0 | 0 | 1.14 | 46.90 | 20.77 | 8.71 | 4.69 | 0 | 0 |
|  | **LTS** | -23.45 | 0 | 0 | 0 | 0 | 0 | 0 | -23.45 | -23.45 | -52.93 | -2.01 | -16.75 | 0 | -5.36 |
|  | **FS** | 0 | 0 | 0 | 0 | 0 | 0 | 0 | -2.68 | -2.68 | -2.68 | -61.64 | 0 | 0 | 0 |
| **L6** | **RS** | 0 | 0 | 0 | 0 | 0 | 0 | 0 | 0.40 | 1.88 | 0 | 0 | 48.78 | 34.17 | 15.41 |
|  | **LTS** | -23.45 | 0 | 0 | 0 | 0 | 0 | 0 | -16.75 | -16.75 | 0 | -5.36 | -23.45 | -66.33 | -8.71 |
|  | **FS** | 0 | 0 | 0 | 0 | 0 | 0 | 0 | 0 | 0 | 0 | 0 | -9.38 | -29.48 | -28.14 |
