# Supplementary material for: Topology, Cross-Frequency, and Same-Frequency Band Interactions Shape the Generation of Phase-Amplitude Coupling in a Neural Mass Model of a Cortical Column
Source: PLoS Comput Biol. 2016 Nov 1;12(11):e1005180. doi: 10.1371/journal.pcbi.1005180 (PMC5089773; doi:10.1371/journal.pcbi.1005180)
Supplement: S3 Table — (DOCX) [file pcbi.1005180.s003.docx]

| **Parameter [units]** | **Interpretation** | **Value** |
| --- | --- | --- |
| $G\left[ mV \right]$ | Gain | $G_{1}=3.25$, $G_{2}=3.25$, $G_{3}=4.00$ |
| $g\left[ s^{-1} \right]$ | Reciprocal of time constant | $g_{1}=330$, $g_{2}=30$, $g_{3}=400$ |
| $\bar{p}\left[ s^{-1} \right]$,$\sigma_{p}\left[ s^{-1} \right]$ | Mean and standard deviation of the external Gaussian input | $\bar{p}=0$ for all populations  $\sigma_{p}=3$ for all populations |
| $b$ | Damping coefficient | $b=0.001$ for all populations |
| $e_{0}\left[ s^{-1} \right]$ | Maximum firing rate | $e_{0}=5$ for all populations |
| $v_{0}\left[ mV \right]$ | Position of the sigmoid function | $v_{0}=6$ for all populations |
| $r\left[ mV^{-1} \right]$ | Steepness of the sigmoid function | $r=0.56$ for all populations |
